# Preparation of biological monolayers for producing high-resolution scanning electron micrographs

**DOI:** 10.1371/journal.pone.0266943

**Published:** 2022-07-08

**Authors:** Shireen Mentor, Franscious Cummings, David Fisher

**Affiliations:** 1 Department of Medical Biosciences, Faculty of Natural Sciences, University of The Western Cape, Cape Town, South Africa; 2 Department of Physics and Astronomy, Electron Microscopy Unit, University of The Western Cape, Cape Town, South Africa; 3 School of Health Professions, University of Missouri, Columbia, Missouri, United States of America; Mohanlal Sukhadia University, INDIA

## Abstract

Scanning electron microscopy (SEM) provides a technical platform for nanoscopic mapping of biological structures. Correct preparation of SEM samples can provide an unprecedented understanding of the nexus between cellular morphology and topography. This comparative study critically examines two coating methods for preparing biological samples for scanning electron microscopy, while also providing novel advice on how to prepare *in vitro* epithelial or endothelial samples for high-resolution scanning-electron microscopy (HR-SEM). Two obstacles often confront the biologist when investigating cellular structures grown under tissue culture conditions, namely., how to prepare and present the biological samples to the HR-SEM microscope without affecting topographical membrane and cellular structural alterations. Firstly, our use of the Millicell cellulose inserts on which to grow our cellular samples in preparation for HR-SEM is both novel and advantageous to comparing the permeability function of cells to their morphological function. Secondly, biological material is often non-conducting, thermally sensitive and fragile and, therefore, needs to be fixed correctly and coated with thin conducting metal to ensure high-resolution detail of samples. Immortalized mouse brain endothelial cells (bEnd5) was used as a basis for describing the preferences in the use of the protocol. We compare two biological sample coating modalities for the visualizing and analysis of texturized, topographical, membranous ultrastructures of brain endothelial cell (BEC) confluent monolayers, namely, carbon and gold:palladium (Au:Pd) sputter coating in preparation for HR-SEM. BEC monolayers sputter-coated with these two modalities produced three-dimensional micrographs which have distinctly different topographical detail from which the nanostructural cellular data can be examined. The two coating methods display differences in the amount of nanoscopic detail that could be resolved in the nanosized membrane cytoarchitecture of BEC monolayers. The micrographical data clearly showed that Au:Pd sputter-coated samples generate descript imagery, providing useful information for profiling membrane nanostructures compared to carbon-coated samples. The recommendations regarding the contrast in two modalities would provide the necessary guidance to biological microscopists in preparing tissue culture samples for HR-SEM.

## Introduction

The successful generation of electron micrographs largely depends on the preparation of the specimen under investigation. Early ultrastructural investigations were limited due to resolution limitations and investigators were restricted in their ability to observe the ultrastructural details of cell membranes and their extracellular topography. This severely limited both the theoretical and experimental approaches to cell biology. HR-SEM allows much higher resolution of the cell’s plasma membrane surface, allowing for the visualization of nanosized morphological structures. Tissue-culture based samples also provide numerous challenges to the microscopists in preparing the samples for HR-SEM. One of the main problems in presenting endothelia or epithelia is that they have to be grown on a biological basement-like material to orientate themselves morphologically into distinct basolateral and apical domains. These domains are both morphologically and functionally different from each other. Therefore, growing the cells on non-physiological surfaces namely, glass or plastic produces endothelial/epithelial cells that are morphological and physiological disorientated. We, therefore, grew our BECs on a cellulose-based insert (Millicell) which allowed our cells to grow into correctly orientated cellular monolayers. These inserts allowed for the measurement of permeability across the monolayer as well as a platform to fix the cells in preparation for HR-SEM.

We further describe two acceptable preparatory coatings of biological tissue and compare the structural differences between the two protocols. For a specimen to yield high-resolution micrographs, the surface of the specimen requires electrical conductivity. Biological material is often non-conducting, thermally sensitive and fragile, therefore, fixation of biological samples must be performed correctly and coated with thin conducting metal such as gold:palladium (Au:Pd) (5 nm), contrary to carbon (15 nm) [1, 2].

**Fig 1:**
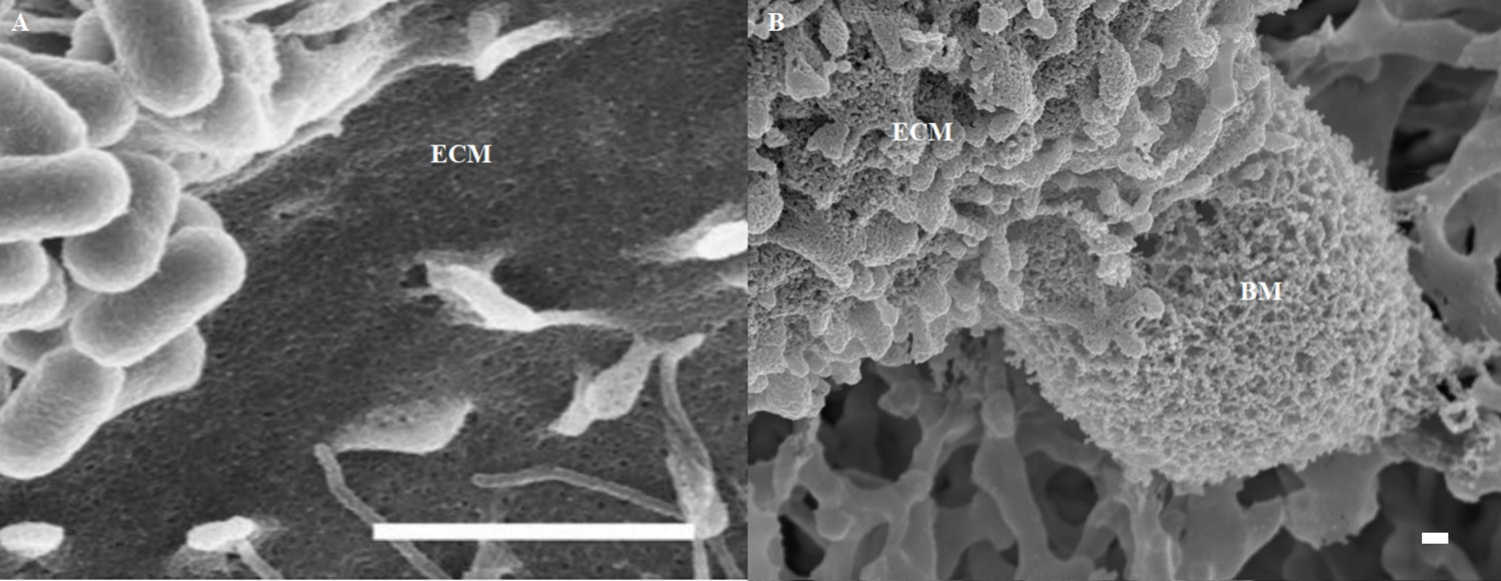
Scanning electron micrographs displaying the apical surface of a polarized epithelial cell interacting with *Escherichia coli* bacteria, grown on gold mesh grids, dried and coated with carbon, scale bar = 2000 nm [3]. **ECM** denotes the epithelial cell membrane (**A**); A polarized brain endothelial cell (bEnd5), grown in our laboratory, on an insert membrane, dried and coated with Au:Pd, scale bar = 2000 nm. **ECM** denotes the endothelial cell membrane and the basement membrane of the BEC is denoted as **BM** (**B**).

We used **Fig 1A and 1B** to illustrate the relative lack of high-resolution detail in a recent HR-SEM micrograph [3] compared with the resolution we routinely are able to generate in our HR-SEM micrographs. **Fig 1A**, depicts a carbon-coated micrograph showing the interaction between correlative light and scanning electron microscopy (CLSEM) of *Enteropathogenic Escherichia coli* (EPEC) with the surface of a polarized epithelial monolayer. The micrograph lacks nanoscaled detail of the ultrastructural profile of an epithelial cell membrane [3]. The preparatory process of this epithelial cell sample may have caused structures to appear obscured and lack three-dimensionality, which could be due to the type of coating material utilized and how excessively it has been applied. Conversely, during monolayer development of the BEC, as seen in **Fig 1B** of a BEC published in a recent study by [4] more nanoscopic details can be observed, as the micrograph displays a detailed plasma membrane surface and the extrusion of an amorphous extracellular matrix, showing molecular details of the plasma membrane, after utilizing the Au:Pd alloy coating material on the BEC membrane. In this methodology paper, we report on the comparison of using both carbon/graphite (C) and Au:Pd coating methods and its ability to yield a greater resolution of ultrastructural detail of the biological sample surfaces /plasma membrane topography.

The electron microscope was utilized due to its ability to generate high-definition (HD) micrographs, at nanometer scaled resolution. In light microscopy, the maximum resolution is approximately 0.2 μm, as opposed to 0.1 mm for the unaided eye (at a standard viewing distance of 25 cm [5]. HR-SEM, on the other hand, enables 3-dimensional (3-D) visualization of biological specimens to a resolution of approximately 10 nm [6]. Therefore, both HR-SEM and HR transmission-EM (HR-TEM) bridge the gaps between resolution produced with light microscopy, which is limited in generating high-resolution micrographs. HR-SEM produces images with a substantial amount of ultrastructural information which illuminates the physical (molecular) composition of a specimen’s surface topography [4, 5]. The *in situ* HR-SEM analysis of BEC monolayer growth was conducted to extrapolate nanostructural dynamics involved in BEC-to-cell interaction. However, upon analysis with carbon-coated samples, we observed large carbon grains which distorted the 3-D nature of the endothelial micrographs. The findings were endorsed in a study conducted by [7] who reported altered morphology of a biological specimen after being coated with carbon. For this comparative study, HR-SEM was employed as a tool for the analysis of the ultrastructural dynamics of the BEC monolayer of the *in vitro* blood-brain barrier (BBB) development. To date, an existing issue that persists in micrographical findings found in the literature to as recent as 2019 is the excessive use of metals applied during sputter coating, thus, it is critical to consider finer and/or lower atomic number elements such as iridium (atomic number = 77) or palladium (atomic number = 46 in contrast to gold (atomic number = 79) [1]. The use of metals with high atomic numbers tend to result in the obscurity of nanoscopic details and, more often than none, biological sample surface appears bulky [1, 3]. Inadequate to poor coating choices for biological sample imaging in HR-SEM remains the current *status quo* for biological sample preparation.

Modern high-resolution microscopy requires a re-evaluation of its current methodological approaches. The objective of this study was to compare two coating modalities to visualize high ultrastructural dynamics of BEC plasma membrane-associated nanostructures. The utilization of an inadequate coating method would lose vital micrographical detail which is a disadvantage to an HR-SEM narrative for describing novel BEC ultrastructures. The detail seen in **Fig 1B** is much more apparent, displaying greater molecular resolution upon coating with Au:Pd (**Fig 1B**), in comparison to carbon (**Fig 1A**). The novelty in this study is not the utilization of a Au:Pd alloy per sé, but in its recommended employment for visualizing ultrastructures, when superficially studying the cell’s plasma membrane surface. The study aims to alert microscopy scientists to make use of the Au:Pd alloy as opposed to non-metal carbon-coating as it obscures a plethora of morphological detail that we can and/or should be observing. The study investigated BEC ultrastructural variation utilizing two coating modalities for HR-SEM studies: (i) metal coating Au:Pd 80:20 application (as opposed to 60:40) and (ii) non-metal, Carbon-coating to decipher between samples exhibited detailed morphological features using two different coating modalities.

## Materials and methods

The protocol described in this peer-reviewed article is published on protocols.io, Updated November 6 2021 dx.doi.org/10.17504/protocols.io.bw37pgrn and is included for printing as S1 File with this article.

### High-resolution scanning electron microscopy

When using HR-SEM, the signal generating the image occurs as a result of the interaction of the primary electron beam with the biological specimen. Briefly, upon interaction, the primary beam electrons induce ionization of the sample’s atoms and the subsequent emission of secondary electrons (SE) from the top-most region of the specimen. The surface-emitted SEs are detected by a scintillator-based Everhart-Thornley detector (ETD), also known as secondary or SE detector. Modern SEMs, however, are more frequently manufactured with in-lens SE detectors, which have the ability to detect fine structures that are invisible to traditional ETDs [8].

For biological materials, which are predominantly hydrocarbons, a low, primary beam energy is desirable to minimize the interaction volume depth in accordance with Eq (1). A small volume allows the operator to study finer specimen surface detail while simultaneously minimizing sample charging caused by secondary electron build-up on the surface. One drawback, however, is a low signal-to-noise ratio caused by the reduced secondary electron emission. This can, however, be solved by coating the sample surface with a thin layer of conducting material such as gold or graphite. The nexus of structural biology is achieving three-dimensionality and investigating the correlation between the morphological framework and its molecular underpinnings. The macromolecular structure is concomitant with its physiology as the shape of any given structure determines its function. Studying both the nanostructural and/or molecular machinery that governs the phenotypic evolution of a BEC unifies our understanding of BBB construction.

The volume created by electron beam when incident on the specimen surface is called the interaction volume and is dependent on the following important parameters: (i) the primary electron beam energy (E_0_), (ii) the average atomic number (Z), (iii) density (ρ) and (iv) average atomic mass (A) of the specimen under investigation [9]. A semi-empirical model of the interaction volume depth, R_p_ is given by [10].


$$R_{p}[in\;\mu m]=\left(\frac{0.0276A}{\rho Z^{0.889}}\right){E_{0}}^{1.67}$$ (1)

In this study, specimens were analyzed using a Zeiss Auriga high-resolution field-emission gun SEM (FEG-SEM), operated at an electron beam energy of 5 keV, a nominal working distance of 5 mm and using an in-lens SE detector for high-resolution imaging.

### Biological sample preparation

The bEnd5 cell line was purchased from the European collection of cell cultures (Sigma-Aldrich, 96091930). The cells were cultured in Dulbecco’s Modified Eagles medium (Whitehead Scientific, Cat no. BE12-719F, South Africa), supplemented with 10% fetal bovine serum (FBS) (Celtic/Biowest, Cat no. S181G-500, South America), 1% *Penicillin/Streptomycin* (Whitehead Scientific, Cat. no. DE17-602E, South Africa), 1% non-essential amino acids (Whitehead Scientific, Cat no. BE13-114E, South Africa) and 1% sodium pyruvate (Whitehead Scientific, Cat no. BE13-115E, South Africa).

bEnd5 Cells were seeded at 5x10^5^ cells/insert on a Millicell, mixed cellulose esters insert membrane (Millipore/Merck, Cat no. PIHA01250, Germany). After exposure of the cell monolayers to the standard culture medium (i.e. supplemented DMEM:F12), at respective time intervals (24-48h), the bEnd5 cells were fixed with 2.5% glutaraldehyde made by adding 10 ml of a 25% solutions of glutaraldehyde (Fluka/ Sigma, Cat no. 49626, Switzerland) in 90 ml of 1X phosphate-buffered saline (PBS) solution (Life Technologies, Cat. no. 20012019, South Africa). Buffers and fixatives used in culture were maintained at pH 7.2 and an osmolality which mimicked that of blood plasma (280–300 mOs/kg) utilizing a Vapor pressure osmometer (VAPRO) (Wescor, South Africa, ser. no. 55201671, Germany), as BECs form the anatomical basis of the brains capillaries and its luminal surfaces are naturally exposed to circulating blood *in vivo*.

#### Chemical fixation

Once bEnd5 monolayers reached confluence the inserts were removed using a lancet and were placed in protein and lipid cross-linking reagent such as 2.5% solution of glutaraldehyde (Fluka/ Sigma-Aldrich) was prepared in standard cell culture buffer- 1X PBS solution [1, 11]. Following a 1 h incubation period at room temperature (RT). The sample could be stored in a 2.5% glutaraldehyde fixative at 4°C overnight. Thereafter, the specimen was washed in 1X PBS (devoid of glutaraldehyde) for 2x5 minutes (min) each. Then samples were washed twice in de-ionized water (H_2_O), each time for 5 min [1].

Biological specimens were removed from the de-ionized H_2_O and placed in a series of graded ethanol (EtOH) (KIMIX, Batch no. 185/11/67 K08/0911) solutions: 50%, 70%, 90%, 95% and twice in 99.9% EtOH for 10 min each. All EtOH solutions were prepared by diluting absolute EtOH in de-ionized H_2_O v/v.

#### Critical point drying

Biological samples are composed largely of H_2_O and sample desiccation using a critical point dryer (CPD) allows for the phased drying of wet, delicate samples from liquid to gas form, by using liquid carbon dioxide (CO_2_) which functions as ‘transitional fluid’. Since H_2_O is not miscible with CO_2_, the alternative is EtOH which serves as the ‘intermediate fluid’/ ‘dehydration fluid.’ Following the dehydration of the fixed samples, it is required that the sample be dry before further processing could occur, this was performed using the Hitachi HCP-2 CPD. Evaporative drying of biological specimens could cause deformation and collapse from the native state of the sample. The deformities in the sample are often due to the surface tension of water, relative to evaporating air [1]. Therefore, CPD was performed by immersing biological samples in liquid with a lower surface tension to air (i.e. CO_2_).

The dehydrated samples were transferred to and retained inside 10 mm diameter aluminium baskets. The basket holders were placed inside the CPD chamber and filled with liquid CO_2_. This step is critical, as the amount of liquid CO_2_ injected into the chamber must be between 50% and 80% of the total chamber volume. The chamber would not reach the critical point (CP) with inadequate liquid CO_2_. The temperature was set to 20°C for 15 min and then 38°C for 5 min until the critical pressure was reached (73 kg/cm^2^).

#### Bio-organic specimen coating for HR-SEM

It is imperative to ensure that the coating material does not compromise the surface details of the specimen during analysis. The coating’s main function is to ensure sufficient surface conductivity, reduction of beam heating and radiation damage, as well as specimen volatility [12]. Post CP drying, the BEC monolayers are coated with either a gold: palladium(Au:Pd) alloy by means of sputter-coating using a Quorum Q150T ES sputter coater, or with a thin layer of carbon during thermal evaporation of a carbon rod using an Emitech K950X carbon coater.

#### Sputter coating using gold:palladium

**Fig 2:**
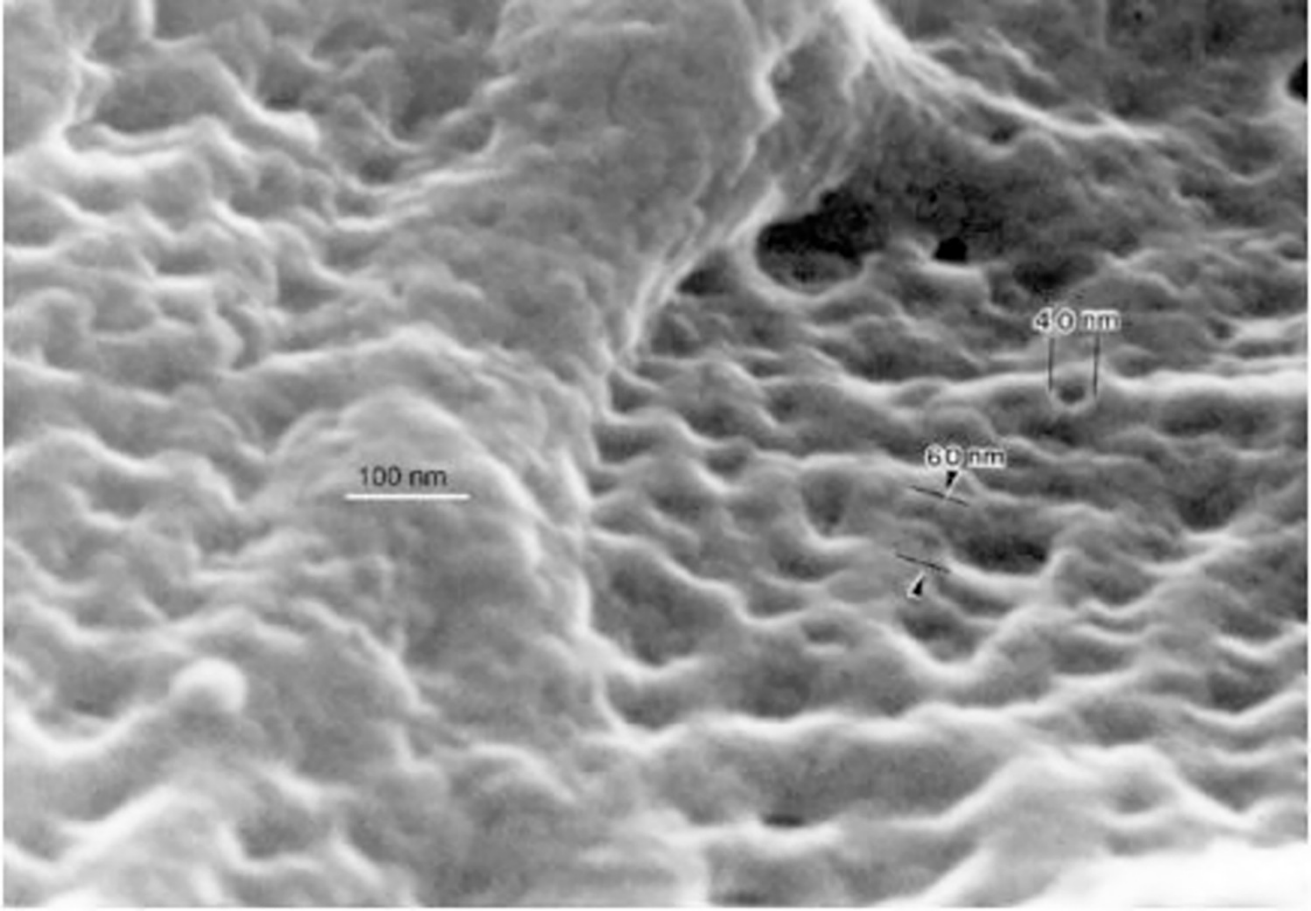
High-resolution SEM of the luminal surface of a capillary coated with Au:Pd [13]. Here the molecular detail on the luminal surface appears obscured, due to bulky coating, thus resulting in loss of ultrastructural detail.

A Au:Pd alloy in an atomic ratio of 80:20 (Au:Pd 80:20) was used as the sputtered target material. The use of Au:Pd 80:20 is much *en vogue* compared to traditional gold only coatings. Palladium prevents Au agglomeration, which is known to produce large islands between 8 nm and 12 nm which result in an uneven coating, especially around the tallest structures in the specimen. This non-homogeneous coating invariably restricts resolution performance [13]. Conversely, Au:Pd 80:20 produces a finer, homogenous film with a particle grain size between 4 nm and 8 nm [14]. This information is supported by a study that involved the imaging of blood capillaries using SEM by [13]. In their study the luminal cell surface of a fenestrated adrenocortical endothelial cell after deposition of a thick metal film of Au:Pd 60:40 is reported, as indicated in the high-resolution micrograph of **Fig 2** [13]. However, although this microgragh shows a fair amount of detail regarding the magnitude of the textured topography of the biological sample, the molecular details are obscured.

For the purpose of this study, the thickness of the Au:Pd 80:20 coating is controlled by sticking to a standard sputtering time of 60 s. A chamber pressure of 10^−5^ mbar is maintained beforehand, with a sputtering pressure of 10^−2^ mbar used during coating. A sputtering current of 40 mA and tooling factor for Au:Pd of 2.3 are used with a quartz, crystal thickness monitor used to measure the thickness of the coating during sputtering. At the above experimental conditions, a deposition rate of roughly 5 nm/min is achievable at a sample-to-target distance of 50 mm. During deposition, the sample stage is rotated at a speed of 70 rotations per minute to ensure even coating across the specimen surface. Based on the above, a nominal coating thickness of 5 nm is thus deposited.

**Fig 3:**
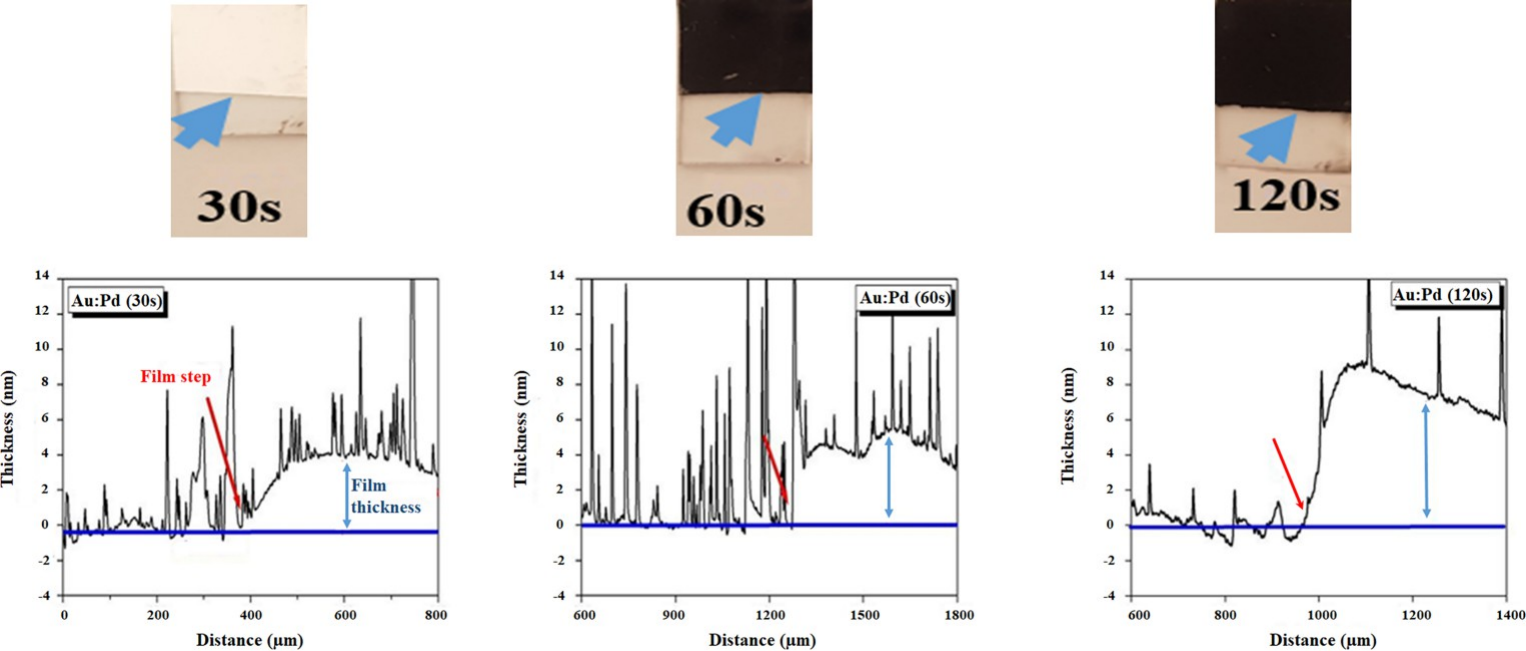
Optical images of the Au:Pd 80:20 coatings sputtered on soda-lime glass for 30 s, 60 s and 120 s (s: Seconds) with correlative profilometry measurements after 30 s, 60 s and 120 s sputter-coated by Au:Pd films approximately 4 nm, 5 nm and 8 nm thick. The glass level, which refers to the soda-lime glass, is indicated by the blue baseline, with the film step and thicknesses indicated by red arrows. The film step refers to the sputter-coated material and the red and blue arrows correspond with each other and indicate the same place where the coating has taken place.

The deposition of layers of material (i.e. metal/non-metal) are denoted as films that range from nanometers to micrometers. Films can be classified into Physical Vapor Deposition (PVD) and Chemical Vapor Deposition (CVD) [15]. To investigate the exact sample thickness, three different sputtering thicknesses are shown in **Fig 3** below. From left to right, Au:Pd layers sputtered on soda-lime glass substrates for 30 s, 60 s and 120 s (s: seconds) are displayed. The reflectivity of the films decreases with increasing sputtering times, readily suggesting an increase in thickness of the films, with the sample deposited at 120 s exhibiting a very dark tinge.

To validate the sputtered thicknesses, surface profilometry was performed using a Veeco Dektak 6M Stylus Profilometer. A diamond stylus tip, with an average diameter of 12.5 μm, was scanned at a step-size of 0.333 μm across the film for a total length of 3 mm. The thickness of the Au:Pd layer was determined by scanning the stylus across the film step, as indicated by the arrows in **Fig 3**.

As shown in **Fig 3,** a sputter time of 30 s produces a film thickness of approximately 4 nm (shown by the red double arrows) which increases to roughly 5 nm after 60 s and 8 nm after 120 s. The spikes observed in the profilometry profiles are due to Au:Pd flakes attaching to the stylus tip. This is very common and indicative of the soft nature of the sputtered film surface. It must be noted that the above results on reasonably flat soda-lime glass produce even coatings, which is not always the case for specimens that are highly textured topographically. Previous results show that coatings of 30 s or less produce specimens that are unevenly coated and still experience surface charging during SEM analysis. Hence, to ensure evenly coated BEC monolayers and to avoid the previously mentioned challenges, a sputter coating time of 60 s (and thus Au:Pd 80:20 coating of 3–5 nm) is recommended for coating biological samples.

#### Thermal evaporation of carbon

**Fig 4:**
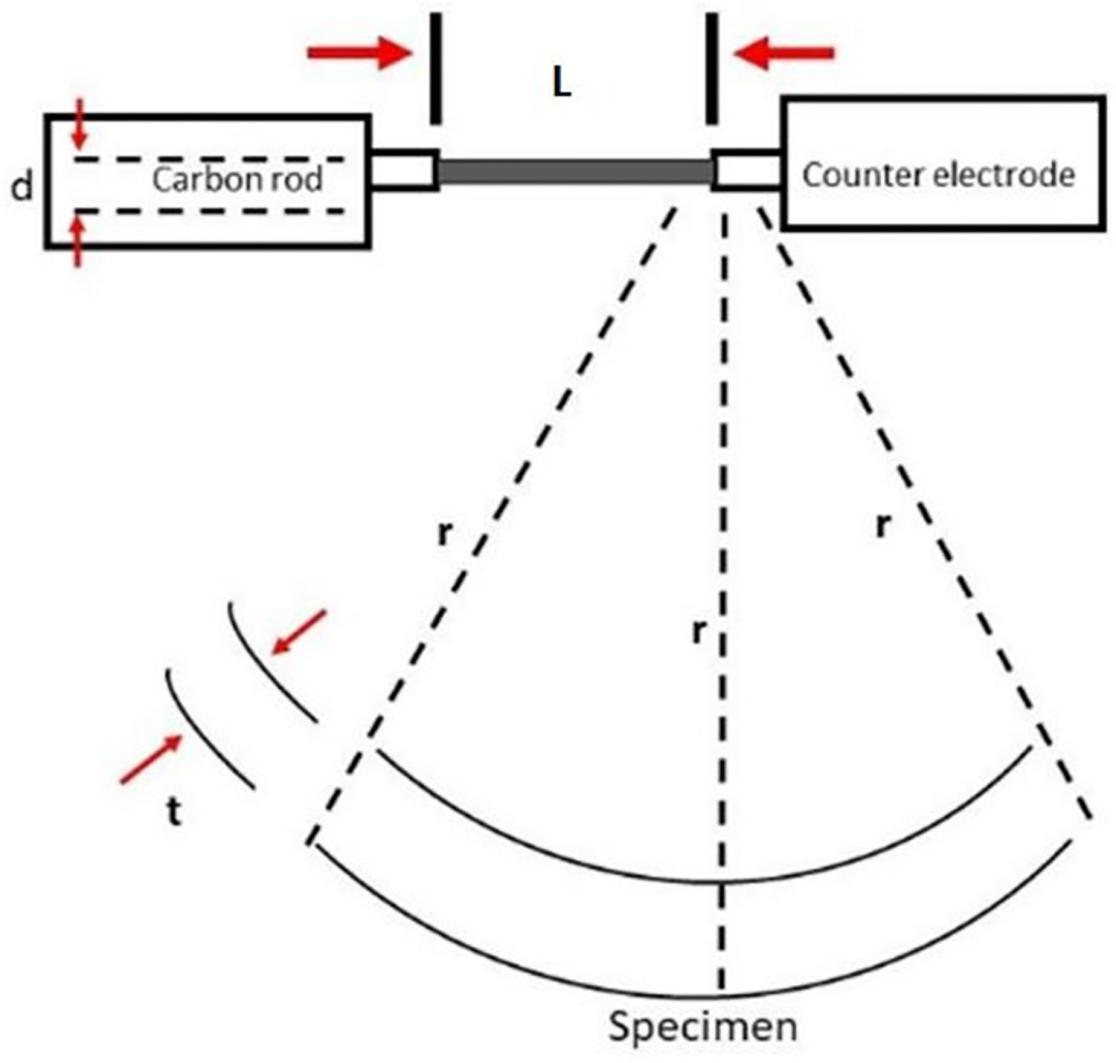
Schematic layout of the thermal evaporation set-up used to deposit carbon layers using the Emitech carbon coater. The *t* symbol denotes the time in seconds, *r* denotes the radius between the rod and the specimen, *d* refers to the diameter of the rod and L denotes the evaporation length.

In comparison, the BEC monolayers were coated with carbon during thermal evaporation. A 3 mm thick carbon rod is sharpened to a diameter (d) of 1.1 mm, with an evaporation length (L) totaling approximately 2 mm. The sharpened rod is mounted against a spring-loaded counter electrode in a vacuum, with the specimen placed a distance, r, from the carbon source. During evaporation, the chamber is pumped down to a vacuum of 10^−3^ mbar, which increases to 10^−1^ mbar for deposition. The current is then slowly increased to a max of 20 A and passed through the carbon rod, thereby heating it beyond the vaporization temperature of carbon; the vaporized carbon plumes subsequently coat the specimen. **Fig 4** shows a schematic representation of this set-up.

**Fig 5:**
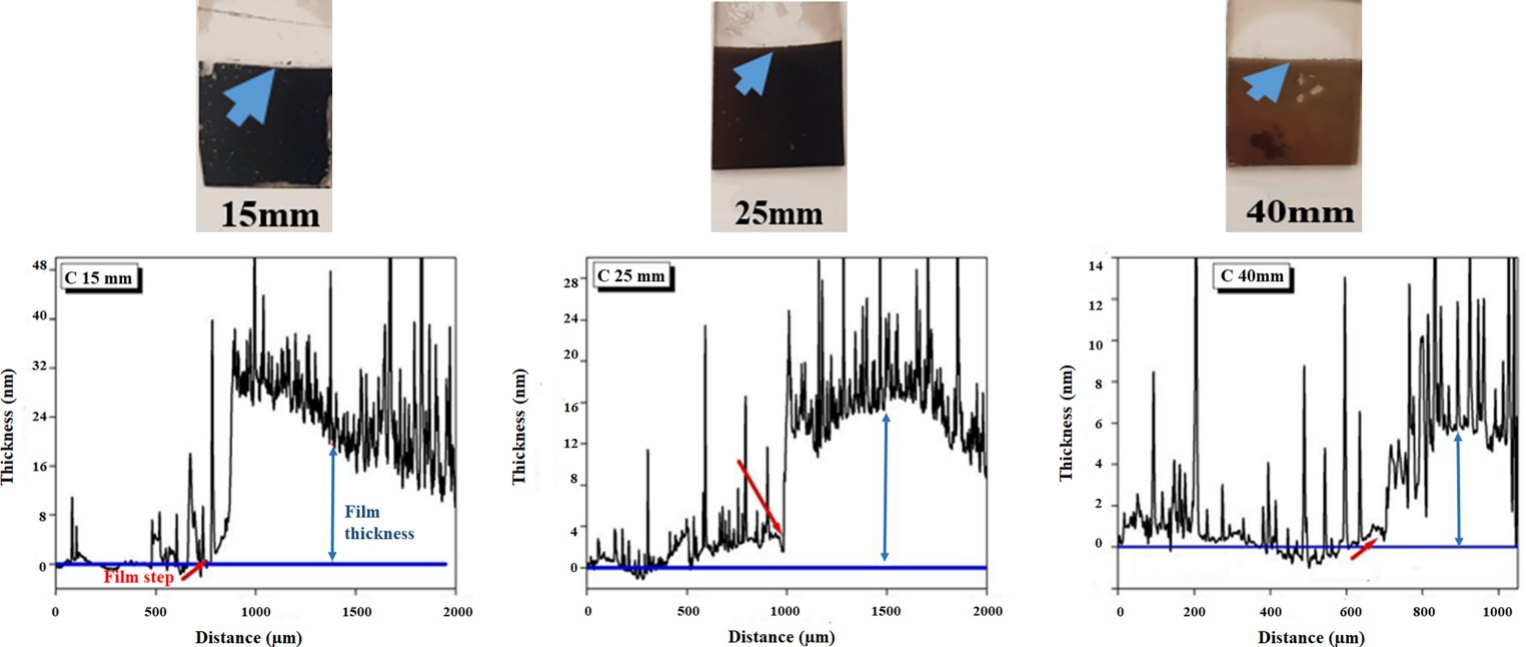
Optical images of carbon coatings deposited on soda-lime glass at a distance of 15, 25 and 40 mm from the carbon source with correlative profilometry measurements of carbon films deposited on soda-lime glass placed 15, 25 and 40 mm from the carbon rod. The glass level is indicated by the blue baseline, with the film step and thicknesses indicated by red arrows. The red and blue arrows correspond with each other, indicating the same place where the coating has taken place.

From **Fig 4**, the average thickness of the deposited carbon film, *t*, can be estimated using simple geometry and the inverse square law as follows:

$$\frac{\pi d^{2}l\rho }{4}=4\pi r^{2}t\rho$$ (2)

or

$$t=\frac{d^{2}l}{{16r}^{2}}$$ (3)

where ρ is the density of the source material. **Fig 5** shows optical images of three carbon films deposited on soda-lime glass. From left to right, the films were deposited at a distance of 15 mm, 25 mm and 40 mm from the carbon source. As shown, the transparency of the films decreases with increasing distance, suggesting a decrease in film thickness. To confirm this, profilometry is once more employed and shown in **Fig 5**.

An average thickness of approximately 20 nm is deduced from the stylus profile of **Fig 5** for the sample placed at 15 mm from the source, which reduces to approximately 16 nm and 6 nm for the 25 mm and 40 mm placed samples, respectively. Closer inspection reveals that the carbon film roughness is more pronounced compared to the Au:Pd 80:20 coatings of **Fig 3** as the spikes in the profiles are more closely spaced and more frequent compared to the Au:Pd coatings. In addition, from the optical images of **Fig 5**, the carbon film integrity is of inferior quality than the Au:Pd films, which had high reflectivity indicative of a compact layer. A simple swab test also reveals that the carbon film is more powdered compared to the metal layers, as they delaminate far easier from the glass slides than the Au:Pd sputtered films. This is to be expected and can be explained by the relative chamber pressures (namely, the metal coatings were deposited in a cleaner chamber (10^−6^ mbar) compared to the carbon coating at a pre-deposition vacuum of (10^−3^ mbar)). Based on the above analysis, a standard sample distance of 25 mm was maintained for all specimens, implying a nominal carbon coating of 16 nm.

## Results and discussion

To date, HREM studies producing 3-D volume images remains scant. The current issue with the loss of resolution, especially with respect to nanoscopic topographical detail, has remained a largely unresolved microscopy problem. Moreover, mapping the membrane pore sizes, nanovesicles and the complex interactive PC spaces of BECs have been thwart with technical difficulty. The absence of these HR-SEM micrographs is a glaring omission in the literature with regards to describing biological surfaces on a nanoscopic level. In parallel, there is a dearth of information regarding the preparation of biological material. Given this lack of crucial preparatory methodology, we utilize the preparation of BECs in monolayers grown on insert cellulose membranes as an exemplar technique. The insert cellulose membrane mimics the basement membrane of epithelia/endothelia. This allows for epithelia or endothelia to express and orientate themselves morphological into distinct basolateral and apical functionality. Our use of these *in vitro* techniques is essential to viewing how cells interact with each other and also cellular interaction within an epi/endothelial monolayer. These techniques provide much greater insight into how these cells would behave within the *in vivo* environment, which is critical for developing *in vitro* models of biological structures to mimic *in vivo* tissues and to further investigate regulatory mechanisms to treat pathological states of disease. The greater the detail that we can observe in cells under normal physiological states the better we can elucidate how these nanoscopic details change within the pathological states. Therefore, the selected and recommended methodology proposed in this paper should tremendously advance the study of biological structures at the nanoscopic level.

### Sputter coating cellular surfaces for high-resolution

The loss of ultrastructural, topographical detail of biological specimens using the incorrect coating modality gives an obscured impression of how these nanostructures are presented within their native state. The incorrect view of a structure would subsequently lead to an incorrect view of its functionality.

**Fig 6:**
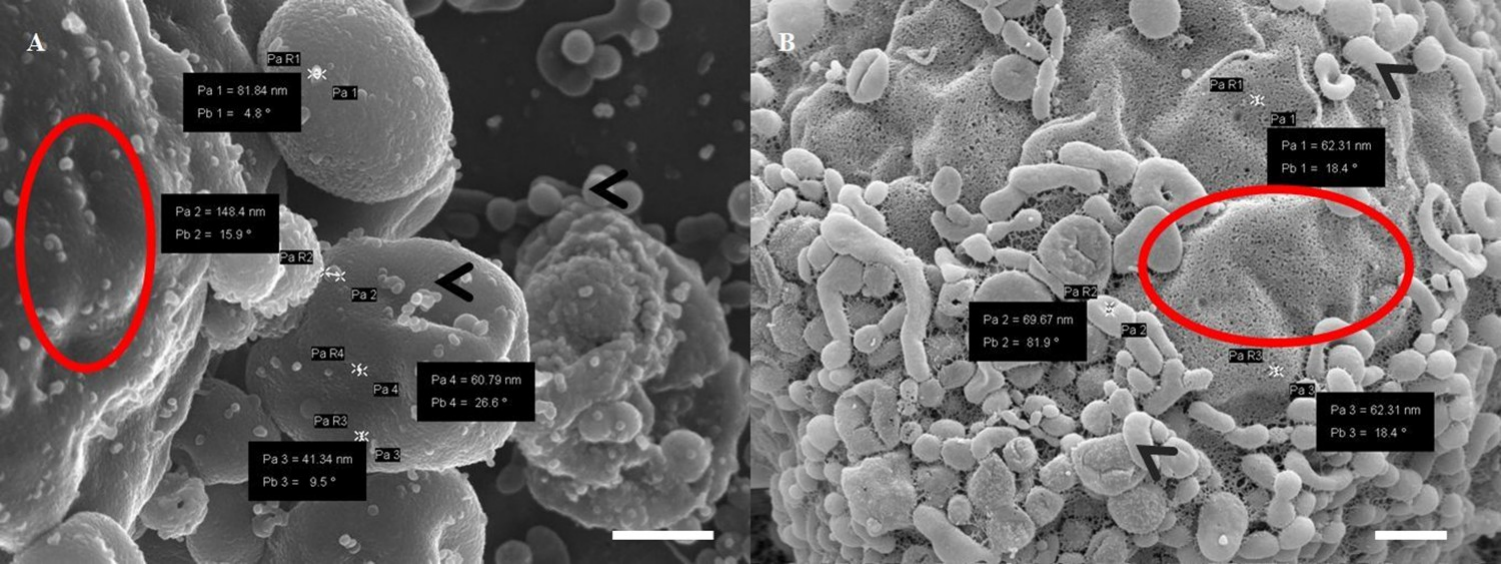
A micrograph representing BEC plasma membrane (PM) surfaces. (A) An image of carbon-coated PM and microvesicle (MV) formation. Scale bar = 1000 nm; (B) An image of a Au:Pd coated PM and vesicle formation. Scale bar = 200 nm. The “red circles” indicate distinct differences in the PM: (A) shows a smooth membrane surface, devoid of pores; (B) displays a porous membrane surface. Pore sizes range between 62–70 nm. The “black arrows” indicate the formation of microvesicles (A) reveals smooth vesicles; (B) exhibits textured vesicles on the PM surface.

We present a series of HR-SEM micrographs to compare two modalities for coating cellular surfaces of monolayers of immortalized mouse brain endothelial cells (bEnd5). In **Fig 6** the topographical data generated between a carbon-carbon coat and a Au:Pd coat show stark differences in ultrastructural definition. **Fig 6B** was able to generate an image that allows for the visualization of detailed features of a phospholipid membrane surface with numerous pore formation and vesicular bodies accrued on the cell membrane surface, compared to **Fig 6A** which shows visible obscurity of HD detail, producing an almost 2-D interpretation of the cell surface.

### Enabling nanoscopic cytoplasmic projections observations

In this study we utilized cytoplasmic projections as an exemplar for this technique, comparing carbon and Au:Pd coated samples to illustrate the discrepancies between the ultrastructural information gathered using a non-metal vs a metal sputter coating modality.

**Fig 7:**
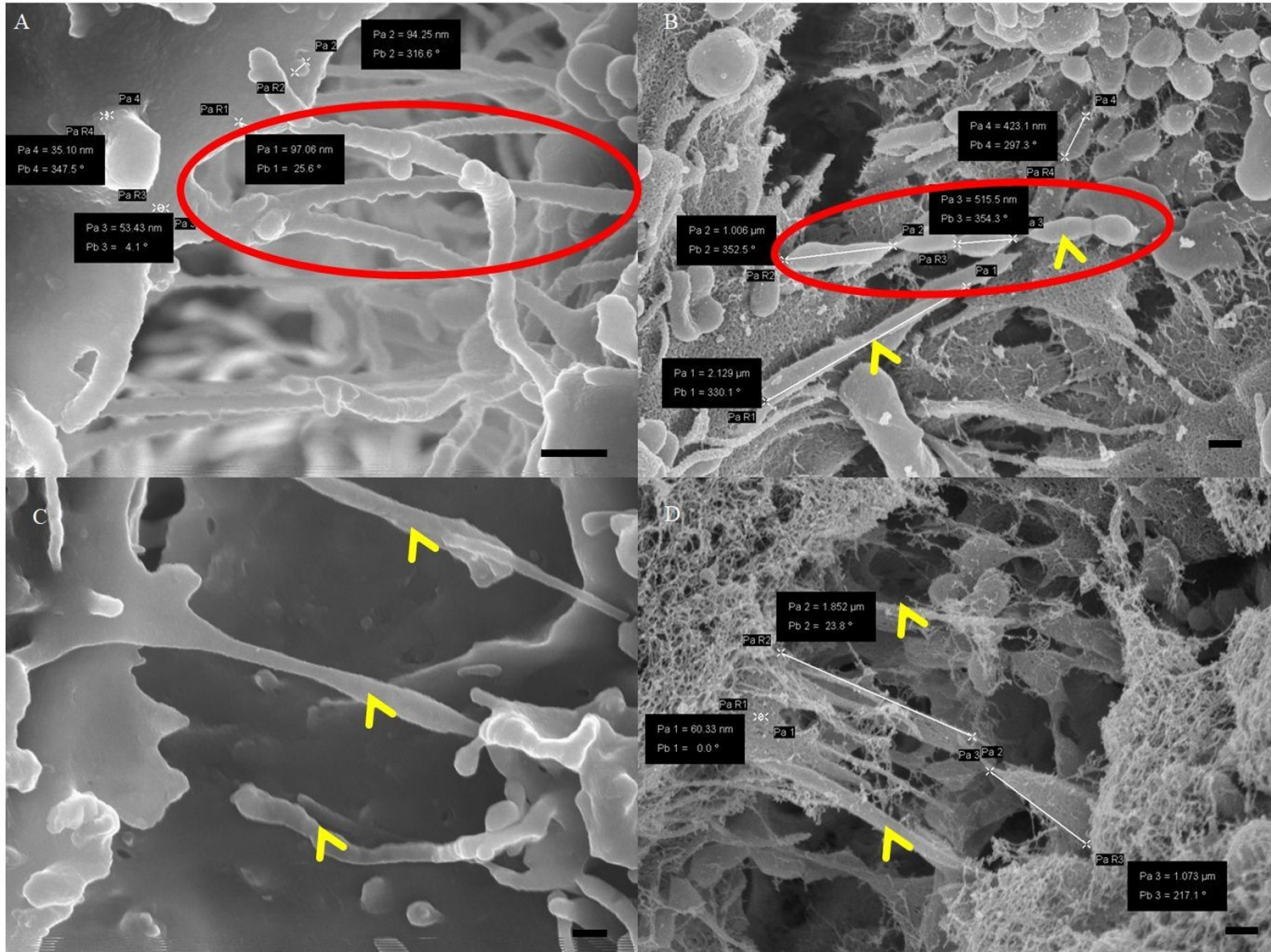
A micrograph representing apicolateral membranous projections between adjacent BECs grown on a Millicell insert membrane. (A) An image of a carbon-coated NT formation, showing smooth, indistinct leading edges, continuous with the cell membrane, see “red circle.” Scale bar = 300 nm; (B) An image of a Au:Pd coated leading edges where more textured lateral vesicles fuse into distinct cytoplasmic projections, see “red circle,” within the PC space, as indicated by the “yellow arrows.” Scale bar = 300 nm; (C) An image of a carbon-coated cytoplasmic projections (CPs) within the PC spaces, see “yellow arrowheads.” Scale bar = 200 nm; (D) An image of Au:Pd coated CPs, indicated by the “yellow arrows.” Scale bar = 300 nm.

**Fig 7A and 7C** (carbon-coated), relative to **Fig 7B and 7D** (Au:Pd coated), shows stark differences in the detailed development of nanotubes (NT) between PC spaces of the BECs. In the carbon-coated micrographs, there are projecting membrane edges that appears continuous with the cell membrane material (**Fig 7B and 7D**). The membrane appears roughly textured and porous, while the projecting NTs are smooth in texture. In addition, extracellular membranous structures visibly suffuse the BEC membrane surfaces in Au:Pd coated samples thus more morphological data is able to be extrapolated from an Au:Pd coating.

### Enabling tight junction interaction observations

When tight junction (TJ) proteins interact between adjacent BECs it forms contact points with its counterparts. These intercellular adherens junctions were, therefore, coated and compared.

**Fig 8:**
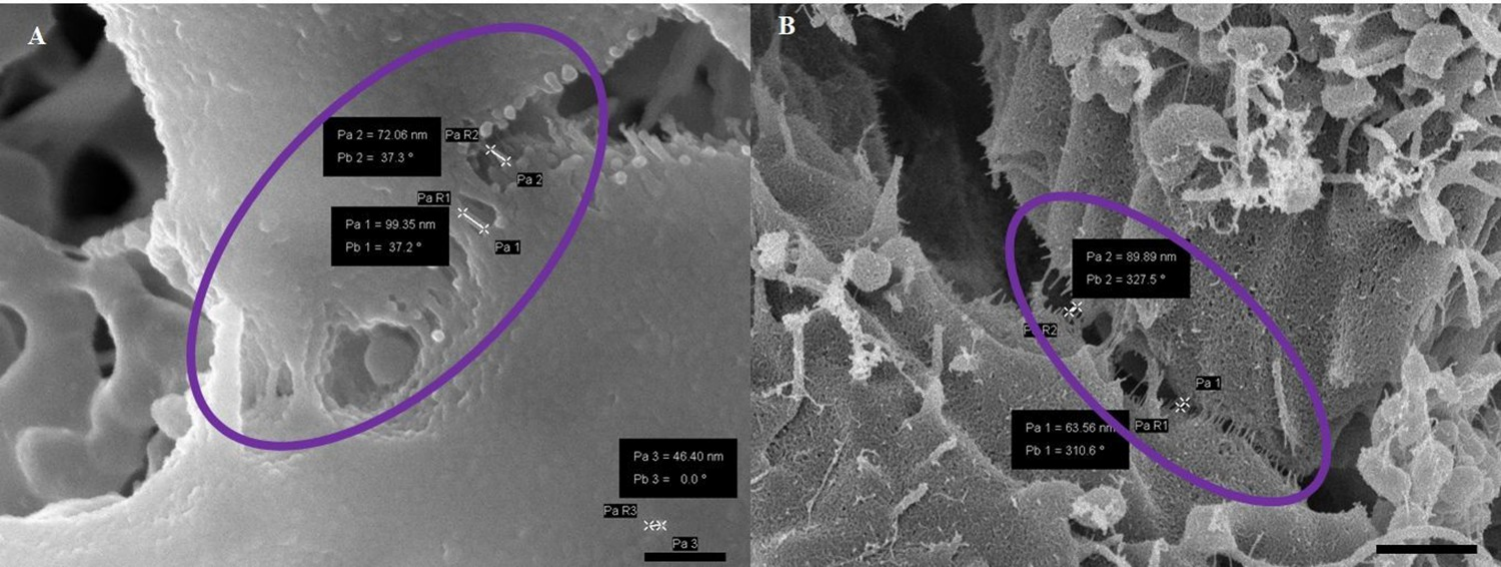
A micrograph representing juxtaposing BEC PMs within the PC spaces. (A) An image of carbon-coated juxtaposed PMs. Scale bar = 1000 nm; (B) An image of Au:Pd coated juxtaposed PMs as seen in the “purple circles”. Scale bar = 1000 nm. (A) When coating with carbon the detail of the membrane is obscured by the coating process, with the membrane presenting an amorphous appearance; (B) shows definitive TJ interaction of two adjacent BECs. This technique provides an unprecedented level of biological detail, making it possible to observe these molecular interactions at the level of the surface of the plasma membrane.

The use of Au:Pd coating in **Fig 8B** clearly demonstrates the interaction of TJs between adjacent BEC membranes, within its PC shunt. Conversely, **Fig 8A** demonstrates that carbon–coating obscures the detailed fusion of adjacent BEC membranes at the paracellular (PC) shunt. In **Fig 8B** the Au:Pd coating enables the visualization of textured surface structures on a nanoscale, such as membranous tent-like leaflets (see “purple circle”) forming what would eventually be overlapping regions across the TJ zones which measured approximately 60–90 nm, occluding the PC shunt.

#### Viewing paracellular spaces under HR-SEM

To comprehend the convoluted nature of the paracellular (PC) spaces between BECs during monolayer establishment, the study attempts to elucidate the complexity of the PC space using carbon coating and Au:Pd.

**Fig 9:**
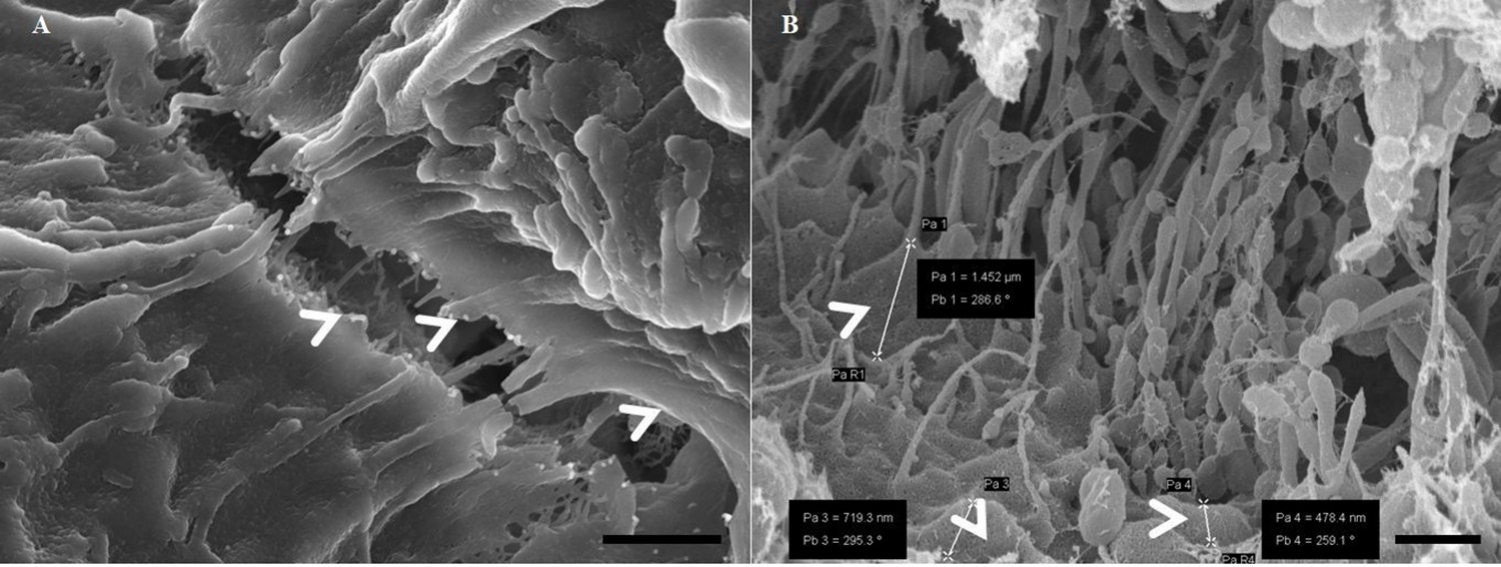
A HR-SEM micrograph representing multiple layers of PM interaction within the PC spaces of BECs. (A) An image of a carbon-coated PM interaction; (B) An image of Au:Pd coated PM interaction as seen by the “white arrowheads.” Scale bar = 1000 nm.

Carbon coating seen in **Fig 9A** produced blunt membrane edges, with a smooth finish of the membranous material, which does not resemble the rich *in situ* biological topography. This is a disadvantage as the morphological data that can be extrapolated remains infinitesimal, thus limiting our understanding of the nanoscaled structural dynamics at play within the PC spaces of BECs. **Fig 9B**, however, exhibits a nuanced, multi-layered PC space enabling the visualization of an array of membranous surface structures, mono-vesicles and multiple vesicular fusion to form nanotubes between adjacent BECs. The Au:Pd, thus, generates more useful, 3-D topographical data that can be investigated and incorporated when describing PC dynamics during BEC monolayer establishment.

Coating the samples with Au:Pd (**Figs 6B–9B**) produced more detailed micrographs, revealing ultrastructural data on the membrane surfaces of BEC monolayers. In addition, Au:Pd prevented charging of images, compared to (**Figs 6A–9A**) which produced a membrane surface that obscured a substantial amount of topographical data. The images generated in this study suggest that coating samples with Au:Pd improved bulk conductivity and generated well contrasted topographical imagery.

To develop high-quality micrographical information, sputter-coating the biological sample with the correct alloy is imperative as it enables the elucidation of ultrastructural, morphological and/or topographical data of a developing BEC in high-resolution. The most frequently used sputter coating material has been gold, iridium, tungsten and carbon which are common choices for low- resolution. Gold particles, however, allow viewing at both low and high magnification [1]. It possesses a high conductivity and relatively small grain size, which makes it ideal for HR imaging. However, the use of Au:Pd, as a metal alloy coating, provides more detail and is advantages in that it provides greater contrast within an HR-SEM micrograph, compared to non-metal carbon coating. Sputter coating with metal alloys are advantages in that it visibly produce greater contrast and HD micrographs and thus it is more beneficial for coating biological samples as it is able to: (i) reduce microscope beam damage, (ii) increase thermal conduction, (iii) reduce sample charging (increased conduction), (iv) improve secondary electron emission, as the metal coat generates higher secondary electrons than samples with no metal/conducting coated (v) reduce beam penetration with improved edge resolution and (vi) protect beam sensitive specimens [12, 14, 16]. It is, thus, critical to ensure that the biological specimen and its environment are modified by the correct sputter-coating. Specimen modification entails coating specimens to increase their conductivity which precludes problems such as electric charge build-up; thermal and radiation damage by the primary beam and thickness of the metallic coating [2, 12]. To retain the biological sample’s authentic characteristics, care should be taken during the fixation process namely: the pH, temperature and osmolality should be within physiologically relevant ranges for the specific cell type. Moreover, strict adherence to timeframes for fixation ensures the preservation of the native state of the biological material under investigation.

The Au:Pd has a significant impact on the quality of the image generated in HD. It illuminates the presence of a porous membrane surface, detailed surface topography, down to individual molecules, jutting out of the cell.

Conversely, the carbon coat obviates analysis of high-resolution, ultrastructural biological data as it reduces the ability to see new, textured nanosized structures during cellular development as it is inclined to produce 2-D planar surfaces and subsequently results in less 3-D anatomizing of the ultrastructures under investigation. HR-SEM utilizing well coated Au:Pd allows for the visualization of a smooth/rough or hollow surface, allows for morphological studies, allows the study of surface texture, whether the structure has pores and pore sizes, normally at a micron size or a nano size.

Coating thickness is a critical aspect to take into account. Based on the profilometry analysis the lowest amount of coating was 15 mm for carbon. The advantage of using Au:Pd is that a coating of a meagre 5 nm is able to produce an even spread of coating over 60 s producing high-resolution ultrastructures which, for the first time, closely presents nanostructures found in its native state.

## Conclusions

When preparing biological samples for HR-SEM imaging the goal is to minimize the aberration of structures found within the native state of the sample. We show that using the tissue-culture technique of growing cellular structures on the cellulose membrane provides novel and optimal experimental conditions for preparing samples for HR-SEM. Given the success we had in elucidating the *in vitro* nanoscopic structure of the endothelial BBB model [4], we have provided a detailed experimental procedure to enhance further investigation of such biological *in vitro* structures (e.g., monolayers of retinal epithelium, germinal epithelium of the blood-testis barrier, endothelial layers of both the systemic and brain endothelia, etc). These structures have not yet been investigated using the methodology we recommend.

Furthermore, the use of Au:Pd sputter-coating, enables the nanostructures of biological specimens to be studied at high-resolution, at a molecular level, whereas carbon-coated samples tend to lose a significant amount of ultrastructural detail. Carbon thermal evaporation may, however, be useful in determining the dimensions of biological structures given its 2-D attributes. In summary, our experimental data recommends using cellulose-based inserts to biologically prepare samples for HR-SEM and Au:Pd sputter-coating as the superior technique that allows for clearly defined nanostructures without adding extrinsic defects, when acquiring HR-SEM nanoscopic topographical details of biological samples. It is recommended that biological material is grown in a manner that mimics the *in situ* situation when preparing it for HR-SEM. This is critical, and if not done correctly, factors such as hypertonicity, osmolality, pH and temperature could result in the severe aberration or/and tearing of tissue/cell samples. We strongly recommend correct coating to preserve the detail of biological samples. Lastly, we recommend Au:Pd sputter-coating, as per our descriptions in the methodology and protocol, to illuminate ultrastructural data in order to study the molecular detail of the BEC membrane surfaces.

## Supporting information

S1 Fig A flow-diagram illustrating the preparation of brain endothelial cell monolayers for imaging using high-resolution electron microcopy. The flow-diagram illustrates the process of monolayer development on a mixed cellulose esters insert membrane, fixation of the BEC monolayer. Fixation was followed by dehydration within a series of graded ethanol concentrations and, thereafter, it underwent critical point drying, replacing ethanol with liquid carbon dioxide at high pressure and regulated temperature until the critical point was reached. After drying, the biological samples were sputter-coated with carbon and Au:Pd in order to ensure the preservation of the sample in its native state when viewed under HR-SEM.
(TIF)

S1 File PDF version of the protocol submitted on protocols io. (PDF)
