# Supplementary figures and images for: Preparation of biological monolayers for producing high-resolution scanning electron micrographs
Source: PLoS One. 2022 Jul 8;17(7):e0266943. doi: 10.1371/journal.pone.0266943 (PMC9269934; doi:10.1371/journal.pone.0266943)

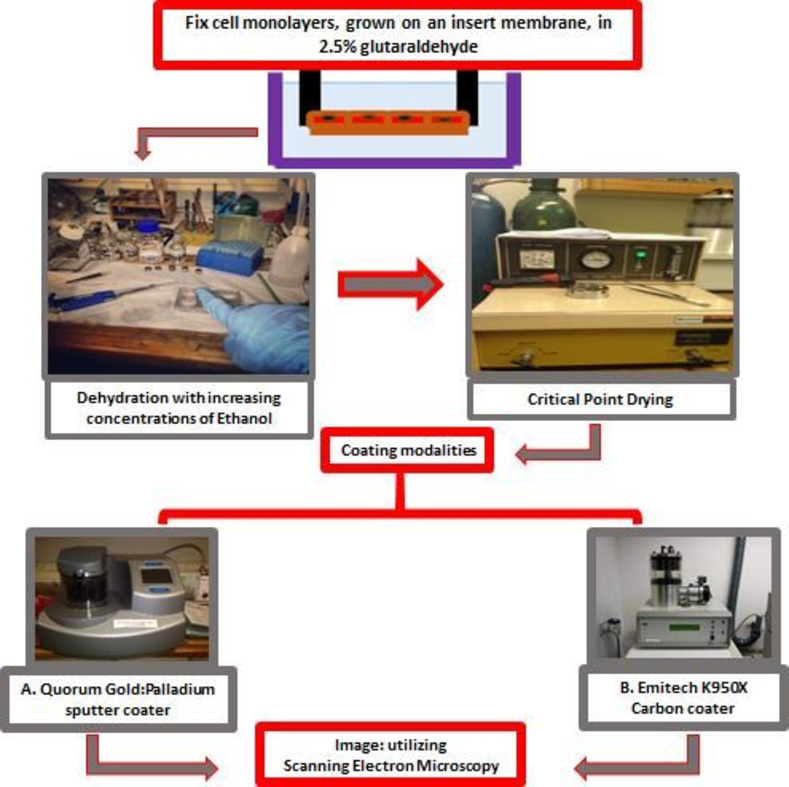

Supplement: S1 Fig — The flow-diagram illustrates the process of monolayer development on a mixed cellulose esters insert membrane, fixation of the BEC monolayer. Fixation was followed by dehydration within a series of graded ethanol concentrations and, thereafter, it underwent critical point drying, replacing ethanol with liquid carbon dioxide at high pressure and regulated temperature until the critical point was reached. After drying, the biological samples were sputter-coated with carbon and Au:Pd in order to ensure the preservation of the sample in its native state when viewed under HR-SEM. (TIF) [file pone.0266943.s001.tif]
