# Supplementary material for: Preparation of biological monolayers for producing high-resolution scanning electron micrographs
Source: PLoS One. 2022 Jul 8;17(7):e0266943. doi: 10.1371/journal.pone.0266943 (PMC9269934; doi:10.1371/journal.pone.0266943)
Supplement: S1 File — (PDF) [file pone.0266943.s002.pdf]

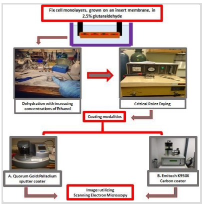

Preparation of biological monolayers for producing high-resolution scanning electron micrographs Code: PLOS2021 [PONE-D-21-26669] - [EMID:f8569bc7b537f6e4]

Shireen Mentor[1], Franscious Cummings[2], David Fisher[1,3]

[1]Department of Medical Biosciences, Neurobiology Research Group, University of the Western Cape, Cape Town, South Africa;

[2]Department of Physics and Astronomy, Electron Microscopy Unit, University of the Western Cape, Cape Town, South Africa;

[3]Adjunct Professor, University of Missouri, School of Health Professions, Columbia, Missouri, United States of America

1 Works for me

shireen.mentor

ABSTRACT

Scanning electron microscopy (SEM) provides a technical platform for nanoscopic mapping of biological structures. Correct preparation of SEM samples can provide an unprecedented understanding of the nexus between cellular morphology and topography. This comparative study critically examines two coating methods for preparing biological samples for scanning electron microscopy, while also providing novel advice on how to prepare *in vitro* epithelial or endothelial samples for high-resolution scanning-electron microscopy (HR-SEM). Two obstacles often confront the biologist when investigating cellular structures grown under tissue culture conditions, viz., how to prepare and present the biological samples to the HR-SEM microscope without affecting topographical membrane and cellular structural alterations. Firstly, our use of the Millicell cellulose inserts on which to grow our cellular samples in preparation for HR-SEM is both novel and advantageous to comparing the permeability function of cells to their morphological function. Secondly, biological material is often non-conducting, thermally sensitive and fragile and, therefore, needs to be fixed correctly and coated with thin conducting metal to ensure high-resolution detail of samples. Immortalized mouse brain endothelial cells (bEnd5) was used as a basis for describing the preferences in the use of the protocol. We compare two biological sample coating modalities for the visualizing and analysis of texturized, topographical, membranous ultrastructures of brain endothelial cell (BEC) confluent monolayers, namely, carbon and gold:palladium (Au:Pd) sputter coating in preparation for HR-SEM. BEC monolayers sputter-coated with these two modalities produced three-dimensional micrographs which have distinctly different topographical detail from which the nanostructural cellular data can be examined. The two coating methods display differences in the amount of nanoscopic detail that could be resolved in the nanosized membrane cytoarchitecture of BEC monolayers. The micrographical data clearly showed that Au:Pd sputter-coated samples generate descript imagery, providing useful information for profiling membrane nanostructures compared to carbon-coated samples. The recommendations regarding the contrast in two modalities would provide the necessary guidance to biological microscopists in preparing tissue culture samples for HR-SEM.

DOCUMENT INFO

Shireen Mentor, Franscious Cummings, David Fisher . Preparation of biological monolayers for producing high-resolution scanning electron micrographs Code: PLOS2021 [PONE-D-21-26669] - [EMID:f8569bc7b537f6e4]. **protocols.io**
https://protocols.io/view/preparation-of-biological-monolayers-for-producin-bw37pgrn

MANUSCRIPT CITATION please remember to cite the following publication along with this document

Mentor S, Cummings F, Fisher D. Preparation of biological monolayers for producing high-resolution scanning electron micrographs. PLOS ONE. 2021.

KEYWORDS

scanning electron microscopy, sputter coating, profilometry, nanostructural cytoarchitecture

IMAGE ATTRIBUTION

Shireen Mentor: Figs 1B and Figs 6-9, including protocol image S1 fig; Franscious Cummings: Figs 3 and 5

CREATED

Aug 03, 2021

LAST MODIFIED

Nov 06, 2021

DOCUMENT INTEGER ID

52063

ABSTRACT

Scanning electron microscopy (SEM) provides a technical platform for nanoscopic mapping of biological structures. Correct preparation of SEM samples can provide an unprecedented understanding of the nexus between cellular morphology and topography. This comparative study critically examines two coating methods for preparing biological samples for scanning electron microscopy, while also providing novel advice on how to prepare *in vitro* epithelial or endothelial samples for high-resolution scanning-electron microscopy (HR-SEM). Two obstacles often confront the biologist when investigating cellular structures grown under tissue culture conditions, viz., how to prepare and present the biological samples to the HR-SEM microscope without affecting topographical membrane and cellular structural alterations. Firstly, our use of the Millicell cellulose inserts on which to grow our cellular samples in preparation for HR-SEM is both novel and advantageous to comparing the permeability function of cells to their morphological function. Secondly, biological material is often non-conducting, thermally sensitive and fragile and, therefore, needs to be fixed correctly and coated with thin conducting metal to ensure high-resolution detail of samples. Immortalized mouse brain endothelial cells (bEnd5) was used as a basis for describing the preferences in the use of the protocol. We compare two biological sample coating modalities for the visualizing and analysis of texturized, topographical, membranous ultrastructures of brain endothelial cell (BEC) confluent monolayers, namely, carbon and gold:palladium (Au:Pd) sputter coating in preparation for HR-SEM. BEC monolayers sputter-coated with these two modalities produced three-dimensional micrographs which have distinctly different topographical detail from which the nanostructural cellular data can be examined. The two coating methods display differences in the amount of nanoscopic detail that could be resolved in the nanosized membrane cytoarchitecture of BEC monolayers. The micrographical data clearly showed that Au:Pd sputter-coated samples generate descript imagery, providing useful information for profiling membrane nanostructures compared to carbon-coated samples. The recommendations regarding the contrast in two modalities would provide the necessary guidance to biological microscopists in preparing tissue culture samples for HR-SEM.

Materials and methods

High-resolution scanning electron microscopy

When using HR-SEM, the signal generating the image occurs as a result of the interaction of the primary electron beam with the biological specimen. Briefly, upon interaction, the primary beam electrons induce ionization of the sample's atoms and the subsequent emission of secondary electrons (SE) from

the top-most region of the specimen. The surface-emitted SEs are detected by a scintillator-based Everhart-Thornley detector (ETD), also known as secondary or SE detector. Modern SEMs, however, are more frequently manufactured with in-lens SE detectors, which have the ability to detect fine structures that are invisible to traditional ETDs (Griffin, 2011).

For biological materials, which are predominantly hydrocarbons, a low primary beam energy is desirable to minimize the interaction volume depth in accordance with equation (1). A small volume allows the operator to study finer specimen surface detail while simultaneously minimizing sample charging caused by secondary electron build-up on the surface. One drawback, however, is a low signal-to-noise ratio caused by the reduced secondary electron emission. This can, however, be solved by coating the sample surface with a thin layer of conducting material such as gold or graphite. The nexus of structural biology is achieving three-dimensionality and investigating the correlation between the morphological framework and its molecular underpinnings. The macromolecular structure is concomitant with its physiology as the shape of any given structure determines its function. Studying both the nanostructural and/or molecular machinery that governs the phenotypic evolution of a brain endothelial cell (BEC) unifies our understanding of blood-brain barrier (BBB) construction.
The volume created by electron beam when incident on the specimen surface is called the interaction volume and is dependent on the following important parameters: (i) the primary electron beam energy (E_0), (ii) the average atomic number (Z), (iii) density (ρ) and (iv) average atomic mass (A) of the specimen under investigation (El Azzouzi *et al.,* 2017). A semi-empirical model of the interaction volume depth, R_p is given by Kanaya and Okayama, (1972).

$$R_p[in\mu m] = (0.0276A/(\rho Z^0.889)) 〖E_0〗^1.67 \quad (1)$$

In this study, specimens were analyzed using a Zeiss Auriga high-resolution field-emission gun SEM (FEG-SEM), operated at an electron beam energy of 5 keV, a nominal working distance of 5mm and using an in-lens SE detector for high-resolution imaging.

Biological sample preparation

Preparation of biological samples

The bEnd5 cell line was purchased from the European collection of cell culture (Sigma-Aldrich, 96091930). The cells were cultured in Dulbecco's Modified Eagles medium (Whitehead Scientific, Cat no. BE12-719F, South Africa), supplemented with 10% fetal bovine serum (FBS) (Celtic/Biowest, Cat no. S181G-500, South America), 1% Penicillin/Streptomycin (Whitehead Scientific, Cat. no. DE17-602E, South Africa), 1% non-essential amino acids (Whitehead Scientific, Cat no.BE13-114E, South Africa) and 1% sodium pyruvate(Whitehead Scientific, Cat no. BE13-115E, South Africa).

bEnd5 Cells were seeded at $5x10^5$cells/insert on a Millicell, mixed cellulose esters insert membrane (Millipore/Merck, Cat no. PIHA01250, Germany). After exposure of the cell monolayers to the standard culture medium (i.e. supplemented DMEM:F12), at respective time intervals (24-48h), the bEnd5 cells were fixed with 2.5% glutaraldehyde made by adding 10 ml of a 25% solutions of glutaraldehyde (Fluka/ Sigma, Cat no. 49626, Switzerland) in 90 ml of 1X phosphate-buffered saline (PBS) solution (Life Technologies, Cat. no.20012019 , South Africa). Buffers and fixatives used in culture were maintained at pH 7.2 and an osmolality which equaled that of blood plasma (280-300 mOs/kg) utilizing a Vapor pressure osmometer (VAPRO) (Wescor, South Africa, ser. no. 55201671, Germany), as BECs form the anatomical basis of the brains capillaries and its luminal surfaces are naturally exposed to circulating blood *in vivo*.

Chemical fixation

Once bEnd5 monolayers reached confluence the inserts were removed using a lancet and were placed in protein and lipid cross-linking reagent such as 2.5% solution of glutaraldehyde (Fluka/ Sigma-Aldrich) was prepared in standard cell culture buffer- 1X PBS solution with an osmolality akin to blood plasma within a range of 280-300 mOsmol/Kg and a pH 7.2 (Faso *et al.,*1994; Fischer *et al.,* 2013). Following a 1h incubation period at room temperature (RT). The sample could be stored in a 2.5% glutaraldehyde fixative at 4°C overnight. Thereafter, the specimen was washed in 1X PBS (devoid of glutaraldehyde) for 2x5 minutes (min) each. Then samples were washed twice in de-ionized H_2O, each time for 5 min (Fischer *et al.,*2013).

Biological specimens were removed from the de-ionized water (H_2O) and placed in a series of graded ethanol (EtOH) (KIMIX, Batch no. 185/11/67 K08/0911) solutions: 50%, 70%, 90%, 95% and twice in 99.9% EtOH for 10 min each. All EtOH solutions were prepared by diluting absolute EtOH in de-ionized H_2O v/v.

Critical point drying

Biological samples are composed largely of H_2O and sample desiccation using a critical point dryer (CPD) allows for the phased drying of wet, delicate samples from liquid to gas form, by using liquid carbon dioxide (CO_2) which functions as 'transitional fluid'. Since H_2O is not miscible with CO_2, the alternative is EtOH which serves as the 'intermediate fluid'/ 'dehydration fluid.' Following the dehydration of the fixed samples, it is required that the sample be dry before further processing could occur, this was performed using the Hitachi HCP-2 CPD. Evaporative drying of biological specimens could cause deformation and collapse from the native state of the sample. The deformities in the sample are often due to the surface tension of water, relative to evaporating air (Fischer *et al.,* 2013). Therefore, CPD was performed by immersing biological samples in liquid with a lower surface tension to air (i.e. CO_2).

The dehydrated samples were transferred to and retained inside 10 mm diameter aluminium baskets. The basket holders were placed inside the CPD chamber and filled with liquid CO_2. This step is critical, as the amount of liquid CO_2 injected into the chamber must be between 50% and 80% of the total chamber volume. The chamber would not reach the critical point (CP) with inadequate liquid CO_2. The temperature was set to 20˚C for 15 min and then 38°C for 5min until the critical pressure was reached (73 kg/cm^2).

Bio-organic specimen coating for HR-SEM

It is imperative to ensure that the coating material does not compromise the surface details of the specimen during analysis. The coating's main function is to ensure sufficient surface conductivity, reduction of beam heating and radiation damage, as well as specimen volatility (Goldstein *et al.,* 1992). Post CP drying, the BEC monolayers are coated with either a gold:palladium (Au:Pd) alloy by means of sputter-coating using a Quorum Q150T ES sputter coater, or with a thin layer of carbon during thermal evaporation of a carbon rod using an Emitech K950X carbon coater.

Sputter coating using gold:palladium

A Au:Pd alloy in an atomic ratio of 80:20 (Au:Pd 80:20) was used as the sputtered target material. The use of Au:Pd 80:20 is much *en vogue* compared to traditional gold only coatings. Palladium prevents Au

agglomeration, which is known to produce large islands between 8 nm and 12 nm which result in an uneven coating, especially around the tallest structures in the specimen. This non-homogeneous coating invariably restricts resolution performance (Zhou *et al.,* 2007). Conversely, Au:Pd 80:20 produces a finer, homogenous film with a particle grain size between 4 nm and 8 nm (Heu *et al.,* 2019). This information is supported by a study that involved the imaging of blood capillaries using SEM by Zhou *et al.,* (2007). In their study the luminal cell surface of a fenestrated adrenocortical endothelial cell after deposition of a 3 nm thick metal film of Au:Pd 60:40 is investigated, as by the high-resolution micrograph of **Fig 2** (Zhou *et al.,* 2007).

For the purpose of this study, the thickness of the Au:Pd 80:20 coating is controlled by sticking to a standard sputtering time of 60 s. A chamber pressure of 10^{-5} mbar is maintained beforehand, with a sputtering pressure of 10^{-2} mbar used during coating. A sputtering current of 40 mA and tooling factor for Au:Pd of 2.3 are used with a quartz, crystal thickness monitor used to measure the thickness of the coating during sputtering. At the above experimental conditions, a deposition rate of roughly 5 nm/min is achievable at a sample-to-target distance of 50 mm. During deposition, the sample stage is rotated at a speed of 70 rotations per minute to ensure even coating across the specimen surface. Based on the above, a nominal coating thickness of 5 nm is thus deposited.

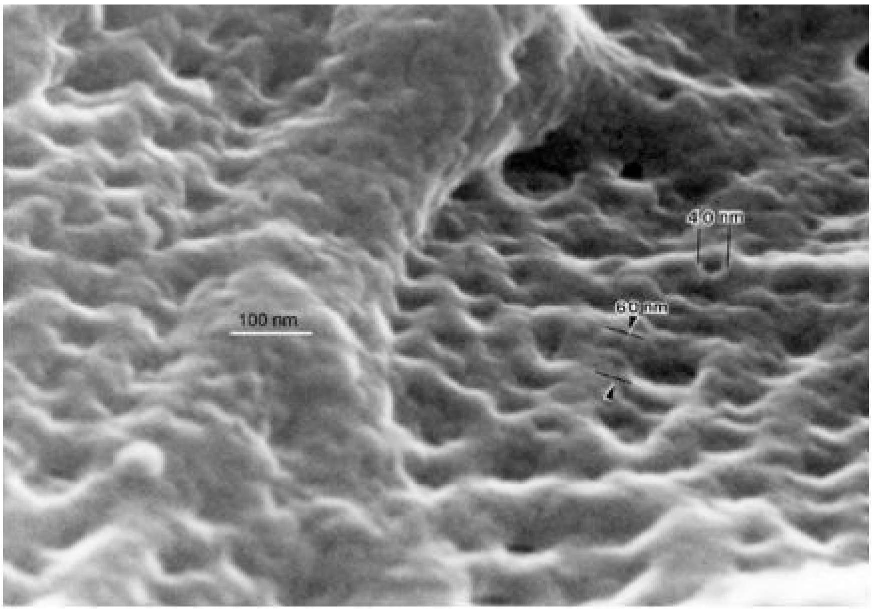

Fig 2. High-resolution SEM of the luminal surface of a capillary coated with Au:Pd (Zhou *et al.,* 2007). Here the molecular detail on the luminal surface appears obscured, due to bulky coating, thus resulting in loss of ultrastructural detail.

The deposition of layers of material (i.e. metal/non-metal) are denoted as films which range from nanometers to micrometers. Films can be classified into two types: (i) Physical Vapor Deposition (PVD) and (ii) Chemical Vapor Deposition (CVD) (Rath, 2014). To investigate the exact sample thickness, three different sputtering thicknesses are shown in **Fig 3** below. From left to right, Au:Pd layers sputtered on soda-lime glass substrates for 30 s, 60 s and 120 s (s: seconds) are displayed. The reflectivity of the films decreases with increasing sputtering times, readily suggesting an increase in thickness of the films, with the sample deposited at 120 s exhibiting a very dark tinge.

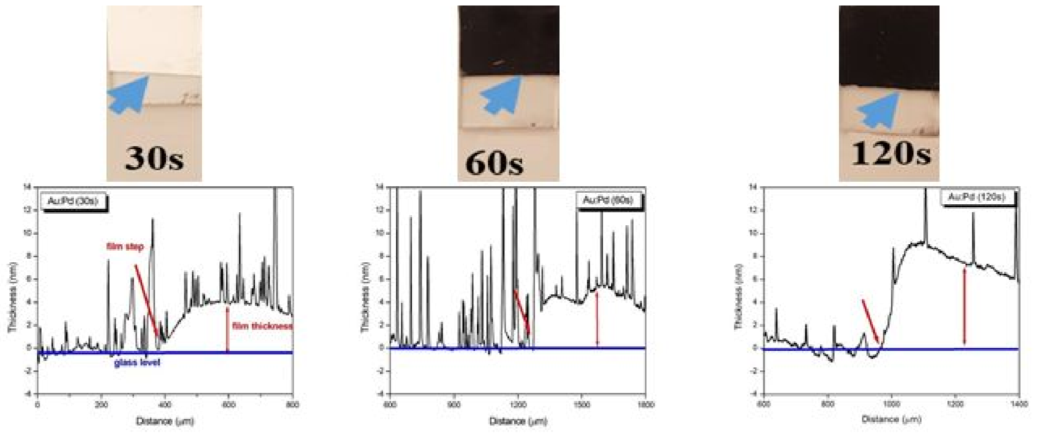

Fig 3. Optical images of the Au:Pd 80:20 coatings sputtered on soda-lime glass for 30, 60 and 120 seconds (S: seconds) with correlative profilometry measurements after 30, 60 and 120 seconds sputter coated by Au:Pd films approximately 4 nm, 5 nm and 8 nm thick. The glass level is indicated by the blue baseline, with the film step and thicknesses indicated by red arrows. The film step refers to the sputter-coated material and the red and blue arrows correspond with each other and indicate the same place where the coating has taken place.

To validate the sputtered thicknesses, surface profilometry was performed using a Veeco Dektak 6M Stylus Profilometer. A diamond stylus tip, with an average diameter of 12.5 µm, was scanned at a step-size of 0.333 µm across the film for a total length of 3 mm. The thickness of the Au:Pd layer was determined by scanning the stylus across the film step, as indicated by the red arrows.

To validate the sputtered thicknesses, surface profilometry was performed using a Veeco Dektak 6M Stylus Profilometer. A diamond stylus tip, with an average diameter of 12.5 µm, was scanned at a step-size of 0.333 µm across the film for a total length of 3 mm. The thickness of the Au:Pd layer was determined by scanning the stylus across the film step, as indicated by the arrows in **Fig 3**.
As shown in **Fig 3,** a sputter time of 30s produces a film thickness of approximately 4 nm (shown by the red double arrows) which increases to roughly 5 nm after 60 s and 8 nm after 120 s. The spikes observed in the profilometry profiles are due to Au:Pd flakes attaching to the stylus tip. This is very common and indicative of the soft nature of the sputtered film surface. It must be noted that the above results on reasonably flat soda-lime glass produce even coatings, which is not always the case for specimens that are highly topographical. Previous results show that coatings of 30 s or less produce specimens that are unevenly coated and still experience surface charging during SEM analysis. Hence, to ensure evenly coated BEC monolayers and to avoid the previously mentioned challenges, a sputter coating time of 60 s (and thus Au:Pd 80:20 coating of 3-5 nm) is recommended for coating biological samples.

Thermal evaporation of carbon
In comparison, the BEC monolayers were coated with carbon during thermal evaporation. A 3 mm thick carbon rod is sharpened to a diameter (d) of 1.1 mm, with an evaporation length (l) totaling approximately 2 mm. The sharpened rod is mounted against a spring-loaded counter electrode in a vacuum, with the specimen placed a distance, r, from the carbon source. During evaporation, the chamber is pumped down to a vacuum of 10^{-3} mbar, which increases to 10^{-1} mbar for deposition. The current is then slowly increased to a max of 20 A and passed through the carbon rod, thereby heating it beyond the vaporization temperature of carbon; the vaporized carbon plumes subsequently coat the specimen. **Fig 4** shows a schematic representation of this set-up.

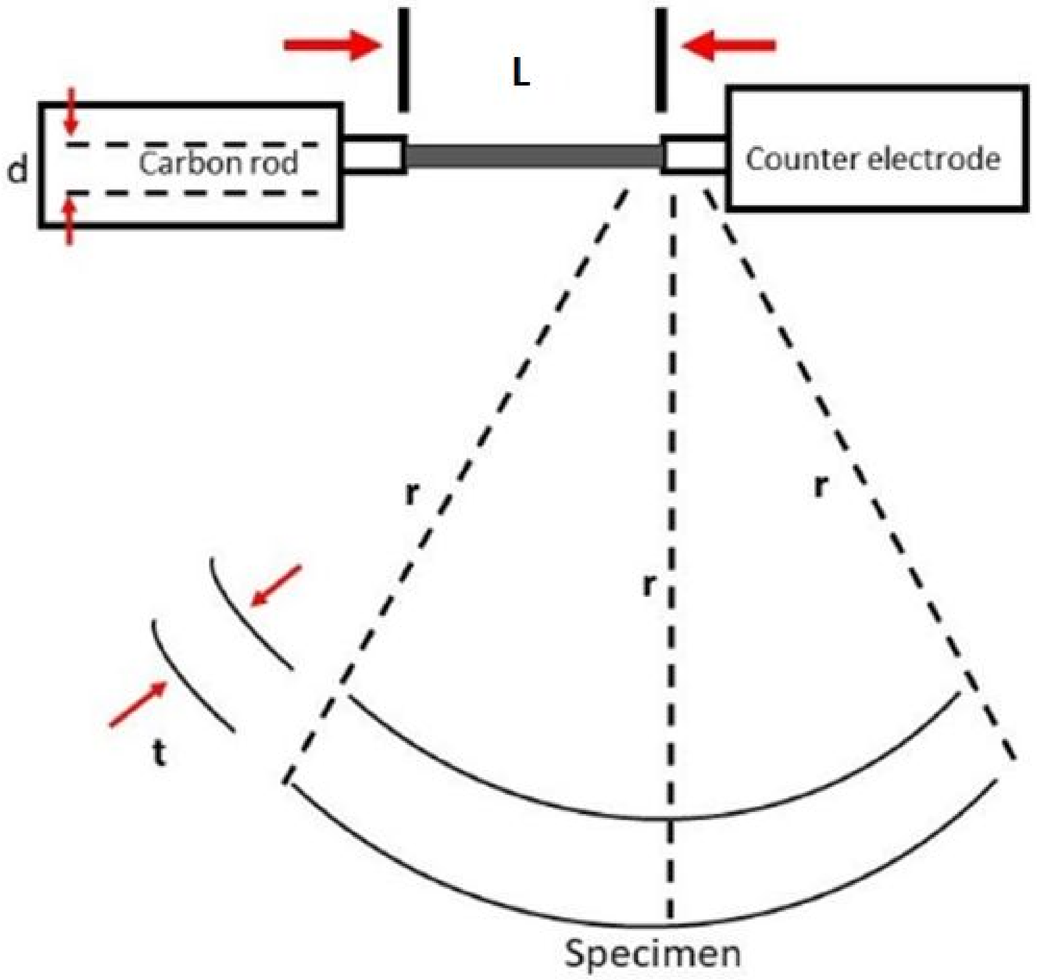

Fig 4. Schematic layout of the thermal evaporation set-up used to deposit carbon layers using the Emitech carbon coater. The *t* symbol denotes the time in seconds, *r* denotes the radius between the rod and the specimen, *d* refers to the diameter of the rod and L denotes the evaporation length.

From **Fig 4**, the average thickness of the deposited carbon film, t, can be estimated using simple geometry and the inverse square law as follows:

$$(\pi d^2 l \rho)/4 = 4\pi r^2 t \rho$$

or $t = (d^2 l)/\llbracket 16r \rrbracket^2$ (2)

where ρ is the density of the source material. **Fig 5** shows optical images of three carbon films deposited on soda-lime glass. From left to right, the films were deposited at a distance of 15 mm, 25 mm and 40 mm from the carbon source. As shown, the transparency of the films decreases with increasing distance, suggesting a decrease in film thickness. To confirm this, profilometry is once more employed and shown in **Fig 5**.

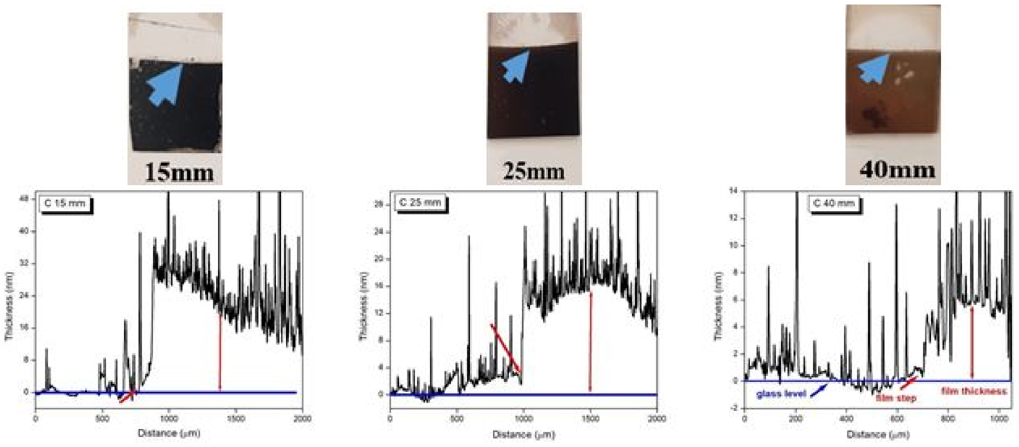

Fig 5. Optical images of carbon coatings deposited on soda-lime glass at a distance of 15, 25 and 40 mm from the carbon source with correlative profilometry measurements of carbon films deposited on soda-lime glass placed 15, 25 and 40 mm from the carbon rod. The glass level is indicated by the blue baseline, with the film step and thicknesses indicated by red arrows. The red and blue arrows correspond with each other, indicating the same place where the coating has taken place.

An average thickness of approximately 20 nm is deduced from the stylus profile of **Fig 5** for the sample placed at 15 mm from the source, which reduces to approximately 16 nm and 6 nm for the 25 mm and 40 mm placed samples, respectively. Closer inspection reveals that the carbon film roughness is more pronounced compared to the Au:Pd 80:20 coatings of **Fig 3** as the spikes in the profiles are more closely spaced and more frequent compared to the Au:Pd coatings. In addition, from the optical images of **Fig 5**, the carbon film integrity is of inferior quality than the Au:Pd films, which had high reflectivity indicative of a compact layer. A simple swab test also reveals that the carbon film is more powdered compared to the metal layers, as they delaminate far easier from the glass slides than the Au:Pd sputtered films. This is to be expected and can be explained by the relative chamber pressures (viz; the metal coatings were deposited in a cleaner chamber (10^{-6} mbar) compared to the carbon coating at a pre-deposition vacuum of (10^{-3} mbar)). Based on the above analysis, a standard sample distance of 25 mm was maintained for all specimens, implying a nominal carbon coating of 16 nm.

Acknowledgements
This work was supported by the infrastructural and academic support provided by the Department of Medical Biosciences at the University of the Western Cape (UWC) and the Electron Microscopy Unit at the University of the Western Cape, Cape Town, South Africa.

References:

El Azzouzi M, Khouchaf L, Achahbar A. Monte Carlo study of the interaction volume changes by the beam skirt in Vp-Sem. *Acta Phys. Pol. A.* 2017; 132: 1393–1398. doi: 10.12693/APhysPolA.132.1393.
Faso L, Trowbridge RS, Quan W, Yao XL, Jenkins EC, Maciulis A, *et al.*. Characterization of a strain of cerebral endothelial cells derived from goat brain which retain their differentiated traits after long-term passage. *Vitr. Cell. Dev. Biol. - Anim.* 1994; 30: 226–235. doi:10.1007/BF02632044.
Fischer ER, Hansen BT, Nair V, Hoyt FH, Dorward DW. Scanning electron microscopy. *Curr Proto in Micro*, 01 May 2012, Chapter 2:Unit 2B.2. Nihms-375012;2013. doi:10.1002/9780471729259.mc02b02s25.
Giurlani W, Berretti E, Innocenti M, Lavacchi A. Measuring the thickness of metal coatings: A review of the methods. *Coatings*. 2020; 10: 1–36. doi:10.3390/coatings10121211.
Goldstein JI, Newbury DE, Echlin P, Joy DC, Romig AD, Lyman CE, *et al.*. Coating and conductivity techniques for SEM and microanalysis. Scanning electron microscopy and X-Ray microanalysis. 1992.

pp 671–740. doi:10.1007/978-1-4613-0491-3_13.
Griffin BJ. A comparison of conventional Everhart-Thornley style and in-lens secondary electron detectors-a further variable in scanning electron microscopy. *Scanning* 2011; 33: 162–173. doi:10.1002/sca.20255.
Heu R, Shahbazmohamadi S, Yorston J, Capeder P. Target material selection for sputter coating of SEM samples. *Micros. Today* 2019; 27: 32–36. doi:10.1017/s1551929519000610.
Kanaya K, Okayama S. Penetration and energy-loss theory of electrons in solid targets. J. Phys. D. Appl. Phys. 1972; 5: 43–58. doi:10.1088/0022-3727/5/1/308.
Kommnick C, Lepper A, Hensel M. Correlative light and scanning electron microscopy (CLSEM) for analysis of bacterial infection of polarized epithelial cells. *Sci. Rep*. 2019; 9: 17079. doi:10.1038/s41598-019-53085-6.
Mentor S, Fisher D. High-resolution insights into the *in vitro* developing blood-brain barrier: Novel morphological features of endothelial nanotube function. *Front. Neuroanat*. 2021; 15: 1–15. doi.org/10.3389/fnana.2021.661065.
Park JB, Kim YJ, Kim SM, Yoo JM, Kim Y, Gorbachev R, *et al.* Non-destructive electron microscopy imaging and analysis of biological samples with graphene coating. *2D Mater*. 3. 2016. doi:10.1088/2053-1583/3/4/045004.
Postek MT, Howard KS, Johnson AH and McMichael KL. Scanning Electron Microscopy: A Student's Handbook, edn. Ladd Research Industries: Postek, M.T. Jr. 1980.
Rath SS. Growth and characterization of gold-palladium thin-films on a silicon substrate. *Ijert*. 2014; 3: 261–264.
Raval N, Maheshwari R, Kalyane D, Youngren-Ortiz SR, Chougule B. and Tekade,R.K. Importance of physicochemical characterization of nanoparticles in pharmaceutical product development. In basic fundamentals of drug delivery,
ISBN 9780128179093, Chpt 10. 2019. pp 369-400. doi: 10.1016/B978-0-12.
Zhou W, Apkarian R, Wang ZL, Joy D. Fundamentals of scanning electron microscopy (SEM). *Scanning Microsc. Nanotechnol. Tech. Appl.* 2007; 1–40. doi:10.1007/978-0-387-39620-0_1.

Web references:

1. Quorum Technologies manual (Auto/Manual High-resolution sputter coater)
Available from: https://www.biologie.uni-hamburg.de/en/service/elektronenmikroskopie/geraete/raster/sputter/sc7640-operating-manual.pdf –[Cited 2021 April 21].

2.Sputter coating brief: Available from: http://www.iitk.ac.in/meesa/SEM/coater_manual_technical.pdf [Cited 21 May 10].
